# CD47 Expression in Classic Hodgkin Lymphoma and Its Association With Tumor Microenvironment

**DOI:** 10.1155/jimr/1680256

**Published:** 2026-01-11

**Authors:** Xiaoyue Xiao, Lin Nong, Yiyang Luo, Jiyan Dong, Xujie Sun, Kang Jiang, Xuemin Xue, Xiaoli Feng

**Affiliations:** ^1^ Department of Pathology, National Cancer Center/National Clinical Research Center for Cancer/Cancer Hospital, Chinese Academy of Medical Sciences and Peking Union Medical College, Beijing, 100021, China, pumc.edu.cn

**Keywords:** CD47, classic Hodgkin lymphoma, immune checkpoints, prognosis

## Abstract

Cluster of differentiation 47 (CD47) has been reported to overexpress in various malignant tumors and is associated with inferior prognosis. However, the critical role of CD47 in classic Hodgkin lymphoma (CHL) and its association with immune checkpoints and tumor microenvironment (TME) remain unclear. Tumor tissues of CHL from Cancer Hospital, Chinese Academy of Medical Sciences and the public dataset from GSE17920 were analyzed. Immunohistochemistry was performed to detect the expression of CD47, programmed cell death protein 1 (PD‐1), and PD ligand 1 (PD‐L1) in CHL. Kaplan–Meier curves and Cox model were used for comparing the clinical outcomes of patients belonging to different subgroups. Bioinformatic analyses included differentially expressed genes (DEGs) screening, functional enrichment, and immune infiltration (CIBERSORT) were performed using GSE17920. In the study, CD47 expression was analyzed in both tumor cells (CD47tumor_score) and the TME (CD47micro_score) of CHL patients. High CD47tumor_score was associated with poorer progression‐free survival (PFS) (*p* = 0.04). Meanwhile, high CD47micro_score was significantly correlated with worse PFS (*p* = 0.02). Univariate Cox regression identified high CD47micro_score as a significant predictor of inferior overall survival (OS; hazard ratio [HR] = 4.91, *p* = 0.03). Additionally, high CD47 expression correlated with increased PD‐L1, and patients with concurrent high CD47 and positive PD‐L1 of tumor cells had the shortest PFS, although this trend was not statistically significant. Importantly, a synergistic prognostic effect between CD47 and PD‐L1 was that patients with high CD47 and/or PD‐L1 of immune cells had significantly worse PFS than those with low expression of both markers. Analysis of public data (GSE17920) confirmed that high CD47 mRNA expression was associated with worse PFS (*p* = 0.02). Functional enrichment analyses revealed that high CD47 expression was linked to pathways involving chemokine signaling and immune cell migration, with increased infiltration of M1 macrophages, neutrophils, and eosinophils in the TME. CD47 might be involved in the disease progression and prognosis of CHL, it had a closely positive correlation with PD‐L1. Targeting of CD47 and PD1/PDL1 might provide a promising strategy in CHL.

## 1. Introduction

Classic Hodgkin lymphoma (CHL) is a highly curable disease, with a 5‐year overall survival (OS) rate of 80%–94% [1]. However, approximately 5%–10% of patients present with primary refractory CHL, and 10%–30% experience relapse or progression after initial treatment [2]. Despite the favorable prognosis, CHL patients are at risk of significant long‐term treatment‐related toxicities, including secondary malignancies, cardiovascular disease, and infertility, which are particularly concerning given that CHL predominantly affects a younger population [3]. Hodgkin–Reed–Sternberg (HRS) cells exhibit high expression of programmed cell death ligand 1 (PD‐L1), with polysomy, copy number gains, or amplifications of the PD‐L1 locus observed in upto 95% of cases, suggesting that this disease may have genetically determined vulnerability to PD protein 1 (PD‐1) blockade [4, 5]. Recently, OS has improved substantially due to the incorporation of brentuximab vedotin (BV) and PD‐1 inhibitors, especially for relapsed or refractory cases [5]. However, the long‐term safety of these agents when combined with chemotherapy remains uncertain [6]. Therefore, ongoing efforts to develop curative strategies with minimal late toxicities and to optimize treatment regimens remain critical focus in the management of CHL.

Cluster of differentiation 47 (CD47) is an immunoglobulin‐like transmembrane protein widely expressed on normal cells. By binding to signal‐regulatory protein alpha (SIRP*α*), it triggers a “don’t eat me” signal that protects cells from phagocytosis [7]. Studies have shown that CD47 is upregulated in various malignancies, enabling immune evasion [8–10]. Increased CD47 expression has been associated with poor prognosis in numerous solid and hematological cancers, including CHL [11–13]. At present, a clinical trial (NCT04788043) studying the combination of CD47 with PD‐1 inhibitor in relapsed/refractory classical HL is ongoing [14]. However, the association of CD47 expression with clinical features in CHL, as well as its relationship with immune checkpoints and tumor‐infiltrating immune cells, remains poorly characterized. Given the central role of PD‐L1 mediated immune evasion in CHL and the emerging understanding of CD47 as an antiphagocytosis signal, we reasoned that HRS cells might exploit these complementary pathways for survival. Therefore, we aimed to investigate their co‐expression and combined prognostic impact, thereby exploring the rationale for dual checkpoint blockade in this disease.

## 2. Methods

### 2.1. Study Population

We initially collected a cohort of 90 CHL patients from the Pathology Department of the Cancer Hospital, Chinese Academy of Medical Sciences (CHCAMS), between 2010 and 2017. After excluding patients with insufficient follow‐up data, incomplete treatment records, or inadequate sample availability, a total of 63 patients were included in the subsequent analyses. Basic clinical characteristics and follow‐up information were retrospectively obtained from the hospital’s electronic medical record system, which provided data on gender, age, histological subtype, Ann Arbor stage, clinical efficacy, and various laboratory test results. Follow‐up information included progression‐free survival (PFS) and OS. The last follow‐up was conducted on November 1, 2024.

### 2.2. Immunohistochemistry and Evaluation

Anti‐CD47 monoclonal antibody (OTI3B10; Zhongshan Golden Bridge), anti‐PD‐L1 monoclonal antibody (22C3, diluted 1:100; DAKO), and anti‐PD1 monoclonal antibody (MXO33, diluted 1:100; Fuzhou Maixin) were used to assess CD47 expression and its correlation with immune checkpoint markers in formalin‐fixed, paraffin‐embedded tissue sections. Immunohistochemical staining was independently evaluated by two experienced pathologists (Lin Nong and Xiaoyue Xiao) who were blinded to the patients’ clinical characteristics and outcomes. The expression of CD47 was quantified using the *H*‐score method [15] in two compartments: (1) in tumor cells (CD47tumor_score), and (2) in the tumor‐infiltrating immune cells (such as lymphocytes, plasma cells, and other mononuclear cells) of the tumor microenvironment (TME) (CD47micro_score). Tumor cells and stromal fibroblasts were explicitly excluded from this assessment. The *H*‐score was calculated as follows: *H*‐score = ∑(percentage [0%–100%] × intensity [1–3]) = (percentage of weakly stained cells × 1) + (percentage of moderately stained cells × 2) + (percentage of strongly stained cells × 3). For PD‐L1 expression, the tumor proportion score (PD‐L1_TPS) and immune positive score (PD‐L1_IPS) were recorded, with a TPS cutoff value of 5% used to define positive staining [16, 17]. For the evaluation of PD‐1 expression, only the IPS was assessed, with a cutoff value of 1% applied to define positive staining [18].

To ensure scoring consistency and minimize bias, the two pathologists initially scored a training set of 30 randomly selected samples together to establish a consensus on the scoring criteria. Thereafter, they evaluated the entire cohort independently and blindly. The inter‐observer reproducibility was then formally assessed on the remaining 60 independent samples using the intraclass correlation coefficient (ICC). The ICC for CD47tumor_score was 0.92 (95% confidence interval [95% CI], 0.87–0.95), for CD47micro_score was 0.85 (95% CI, 0.84–0.94), and for PD‐L1_IPS was 0.87 (95% CI, 0.79–0.92), indicating excellent agreement. For the cases where the scores differed by more than 10 points, a third senior pathologist (Xuemin Xue) adjudicated to obtain a final score.

### 2.3. Public Dataset Analysis

The independent public dataset GSE17920, obtained from the Gene Expression Omnibus (GEO) (https://www.ncbi.nlm.nih.gov/geo/), comprising 130 CHL patients, was included in this study. The optimal cutoff point for CD47 mRNA expression in the dataset was determined using the R package “maxstat” (version 0.7–25). Differentially expressed genes (DEGs) were identified using the “limma” package (version 3.56.2) in R, with thresholds set at |log_2_ fold change| > 1 and adjusted *p*‐value <0.05. Gene Ontology (GO) enrichment analysis and Kyoto Encyclopedia of Genes and Genomes (KEGG) pathway analysis were conducted using the “clusterProfiler” package (version 4.8.3) to investigate the biological functions and pathways associated with the DEGs. Data visualization was performed using R packages, including “ggplot2” (version 3.5.1) and “enrichplot” (version 1.20.3). Immune cell infiltration in CHL was analyzed using CIBERSORT, as previously described [19].

### 2.4. Statistical Analysis

Statistical analyses were performed by using MedCalc (version 15.8.0), IBM SPSS Statistics (version 21), and R software (version 4.3.0). Cutoff values for classifying CD47tumor_score, CD47micro_score and PD‐L1_IPS were determined using the “maxstat” R package (version 0.7–25), with detailed cutoff points provided in Table S1. Group comparisons were conducted using the chi‐square test and Fisher’s exact test. The correlation between two variables was assessed using Spearman’s correlation test. Survival correlations were evaluated through Kaplan–Meier curves, log‐rank tests, and univariate and multivariate Cox proportional hazards analyses.

## 3. Results

### 3.1. Patients’ Characteristics

In this study, 63 patients from the CHCAMS cohort and 130 patients from the publicly available British Columbia Cancer Agency (BCCA) cohort were included. The study flowchart was presented in Figure 1. The baseline characteristics of the enrolled patients from the CHCAMS cohort were comparable to those of their respective original cohorts, as summarized in Table 1. Approximately 17.5% of patients presented with B symptoms, and the majority were diagnosed with early‐stage disease (Ann Arbor stage I/II, *n* = 44, 69.8%). Most patients received doxorubicin, bleomycin, vincristine, and dacarbazine (ABVD) as their first‐line chemotherapy regimen. One patient was treated with cyclophosphamide, hydroxydaunorubicin, etoposide, and prednisone (CHEP), while 13 patients received bleomycin, etoposide, doxorubicin, cyclophosphamide, vincristine, procarbazine, and prednisone (BEACOPP). The median follow‐up duration was 9.77 years (range, 0.38–15.58 years). The 5‐year OS rate was 85.7%, while the 5‐year PFS rate was 71.4%.

Figure 1Flowchart of study participants internal cohort (A) and external cohort (B). CHCAMS, Cancer Hospital, Chinese Academy of Medical Sciences; BCCA, British Columbia Cancer Agency.

**Figure:**
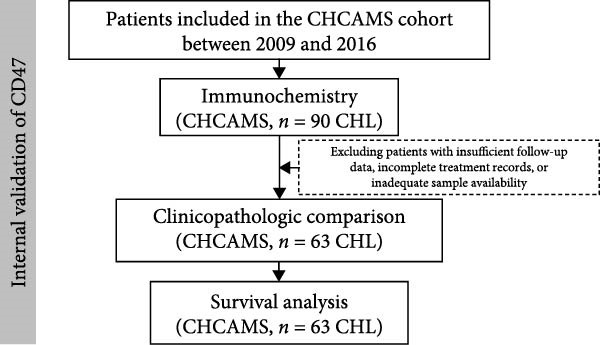
(A)

**Figure:**
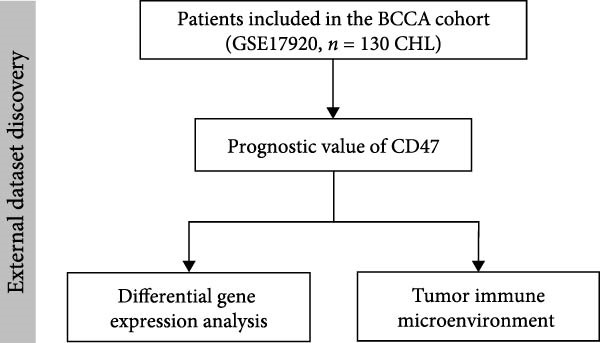
(B)

**Table 1:** The baseline characteristics of the enrolled patients.

Characteristics	Complete CHCAMS cohort *n* = 90	Current CHCAMS cohort *n* = 63
Gender (%)		
Male	54 (60.0)	38 (60.3)
Female	36 (40.0)	25 (39.7)
Age (years)		
Mean	35	35
Range	11–77	11–77
^a^Histological subtypes (%)		
Nodular sclerosis	54 (60.0)	36 (57.1)
Mixed cellularity	22 (24.4)	16 (25.4)
Lymphocyte rich	5 (5.6)	3 (4.8)
Lymphocyte depleted	2 (2.2)	2 (3.2)
EBER (%)		
Positive	19 (21.1)	16 (25.4)
Negative	71 (78.9)	47 (74.6)
^a^Ann Arbor stage (%)		
Early stage (I–II)	62 (68.9)	44 (69.8)
Advanced stage (III–IV)	19 (21.1)	13 (20.6)
^a^First‐line treatment (%)		
ABVD	65 (72.2)	45 (71.4)
BEACOPP	16 (17.8)	13 (20.6)
Others	3 (3.3)	1 (1.6)
^a^Clinical efficacy (%)		
CR	25 (27.8)	17 (27.0)
Non‐CR	47 (52.2)	35 (55.6)

^a^The item had missing cases.

### 3.2. Clinicopathologic Comparison

**Figure 2:**
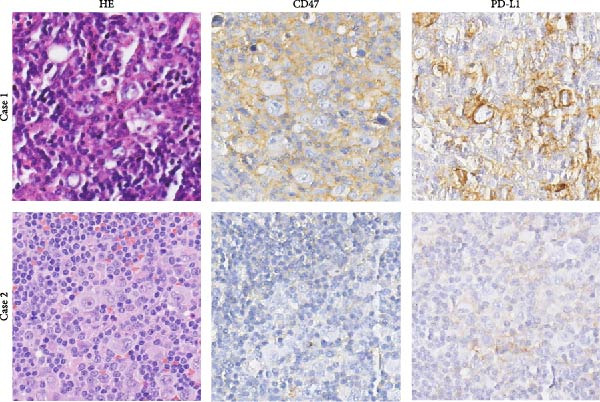
Representative images of CD47 and PD‐L1 by immunohistochemistry. Case 1 represents the patient with positive expression of CD47 and PD‐L1, and case 2 represents the patient with negative expression of CD47 and PD‐L1.

To investigate the expression patterns of CD47 and immune checkpoints, immunohistochemistry was performed using the corresponding antibodies in the CHCAMS cohort. Representative images are presented in Figure 2. To comprehensively analyze the similarities and differences in CD47 expression at both the tumor and TME levels in CHL, separate evaluations were conducted (Table 2). At both the tumor and TME levels, no statistically significant associations were observed between CD47 expression and clinical stage, histological subtypes, sex, EBV status, presence of B symptoms, IPS of PD1, or any laboratory tests. However, patients with high CD47 expression on HRS cells were less likely to achieve the complete response (CR) (*p* = 0.016), but more easily exhibited positive TPS (*p* = 0.012) and higher IPS (*p* = 0.008) of PD‐L1. High CD47 expression in the TME was also associated with positive TPS (*p* = 0.008) and increased IPS (*p* = 0.011) of PD‐L1. Notably, a significant correlation was observed between the CD47tumor_score and the CD47mciro_score (*r* = 0.65, *p* < 0.0001, Figure S1).

**Table 2:** Relation between CD47 expression and clinicopathological characteristics.

Variables	CD47tumor_score		*p*‐Value	CD47mirco_score		*p*‐Value
	High *n* = 43	Low *n* = 20		High *n* = 44	Low *n* = 19	
Age (mean, years)	39	28	0.952	38	30	0.490
Gender	^—^	—	0.090	—	—	0.167
Male	29	9	—	29	9	—
Female	14	11	—	15	10	—
^a^B symptom	—	—	0.984	—	—	1.000
Absence	32	14	—	32	14	—
Presence	7	4	—	8	3	—
^a^Histological subtypes	—	—	0.244	—	—	0.147
Nodular sclerosis	23	13	—	24	12	—
Mixed cellularity	14	2	—	14	2	—
Lymphocyte rich	2	1	—	1	2	—
Lymphocyte depleted	2	0	—	2	0	—
EBER	—	—	0.196	—	—	0.837
Negative	30	17	—	32	15	—
Positive	13	3	—	12	4	—
^a^Ann Arbor stage	—	—	0.276	—	—	0.101
Early stage (I–II)	28	16	—	28	16	—
Advanced stage (III–IV)	11	2	—	12	1	—
^a^Clinical efficacy	—	—	**0.016**	—	—	0.090
CR	8	9	—	9	8	—
Non‐CR	28	7	—	28	7	—
^a^ALB (g/L)	—	—	0.399	—	—	0.191
<40	12	4	—	13	3	—
≥40	24	14	—	24	14	—
^a^HGB ( g/L)	—	—	1.000	—	—	1.000
<105	1	1	—	1	1	—
≥105	40	18	—	41	17	—
^a^WBC (G/L)	—	—	0.620	—	—	0.059
<15	33	17	—	32	18	—
≥15	8	2	—	10	0	—
^a^LYC (G/L)	—	—	1.000	—	—	1.000
<0.6	1	1	—	1	1	—
≥0.6	36	18	—	37	17	—
PD‐L1_TPS	—	—	**0.012**	—	—	**0.008**
Negative	4	7	—	4	7	—
Positive	39	13	—	40	12	—
PD‐L1_IPS	—	—	**0.008**	—	—	**0.011**
Low	19	16	—	20	15	—
High	24	4	—	24	4	—
PD1_IPS	—	—	0.858	—	—	0.508
Negative	32	16	—	32	16	—
Positive	11	4	—	12	3	—
CD47tumor_score	—	—	—	—	—	**<0.001**
Low	—	—	—	3	17	—
High	—	—	—	41	2	—
CD47micro_score	—	—	**<0.001**	—	—	—
Low	2	17	—	—	—	—
High	41	3	—	—	—	—

*Note:* Bold indicates statistical significance.

^a^The item had missing cases.

### 3.3. Survival Analysis

We applied Kaplan–Meier curves and the log‐rank test to compare OS and PFS between CD47^low^ and CD47^high^ patients at both the tumor and TME levels in the CHCAMS cohort (Figure 3). Patients with a high CD47tumor_score (*n* = 42) had significantly worse PFS (*p* = 0.04) compared to those with a low CD47tumor_score (*n* = 20); however, no significant difference in OS was observed (*p* = 0.80). Similarly, patients with a high CD47micro_score (*n* = 42) exhibited poorer PFS (*p* = 0.02) and the unfavorable OS trend (*p* = 0.40). Notably, a high CD47micro_score was the only significant predictor of worse OS in univariate Cox regression analysis (hazard ratio [HR] = 4.91; 95% CI: 1.15–21.04, *p* = 0.033), although its impact on PFS did not reach statistical significance (Table S2). Limited by sample size, no statistically significant markers were identified in multivariable analyses. We then evaluated the prognostic value of immune checkpoints (PD‐1/PD‐L1) (Figure S2). Among these, only patients with a high PD‐L1_IPS showed significantly worse PFS (*p* = 0.03), while a trend toward worse OS was observed but did not reach statistical significance (*p* = 0.45). For patients with a positive PD‐L1_TPS score, trends toward poorer OS and PFS were also observed, though these did not achieve statistical significance. No significant prognostic difference was observed between patients with negative and positive PD‐1_IPS. Further, we evaluated the synergistic effect of CD47 and PD‐L1 across different cellular compartments (Figure 4). Patients with both high CD47tumor_score and positive PD‐L1_TPS showed a nonsignificant trend toward worse PFS and OS compared to all other patients. A more pronounced effect was observed when analyzing the combination of CD47micro_score and PD‐L1_IPS, which revealed a synergistic effect on prognosis, patients with at least one marker in the high‐expression group had significantly worse PFS compared to those with low CD47micro_score and low PD‐L1_IPS.

Figure 3Survival analysis of CD47 expression in the CHCAMS cohort. (A) Kaplan–Meier curves of PFS according to CD47tumor_score. (B) Kaplan–Meier curves of OS according to CD47tumor_score. (C) Kaplan–Meier curves of PFS according to CD47micro_score. (D) Kaplan–Meier curves of OS according to CD47micro_score.

**Figure:**
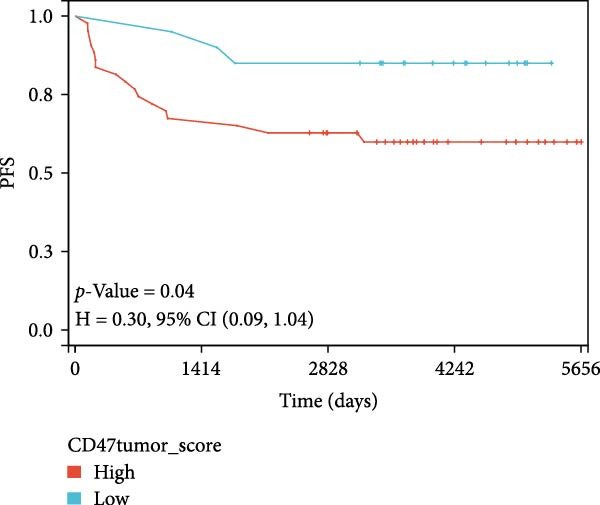
(A)

**Figure:**
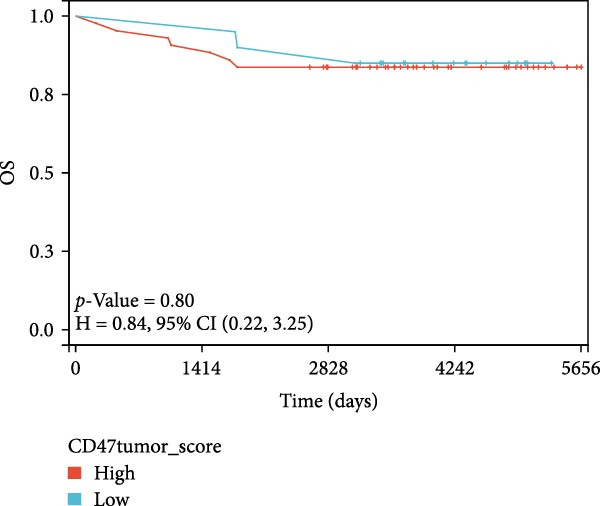
(B)

**Figure:**
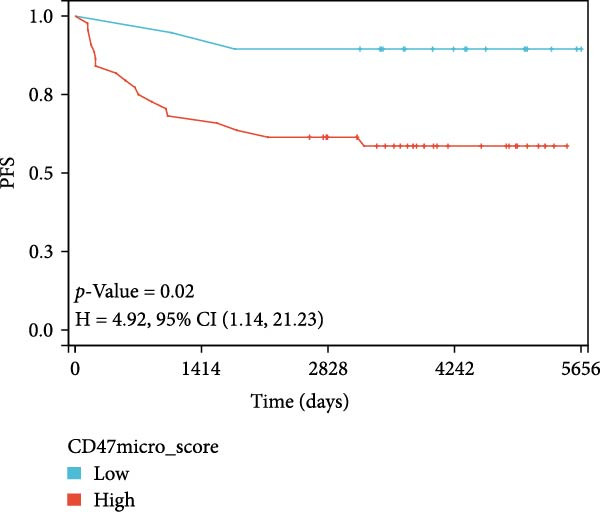
(C)

**Figure:**
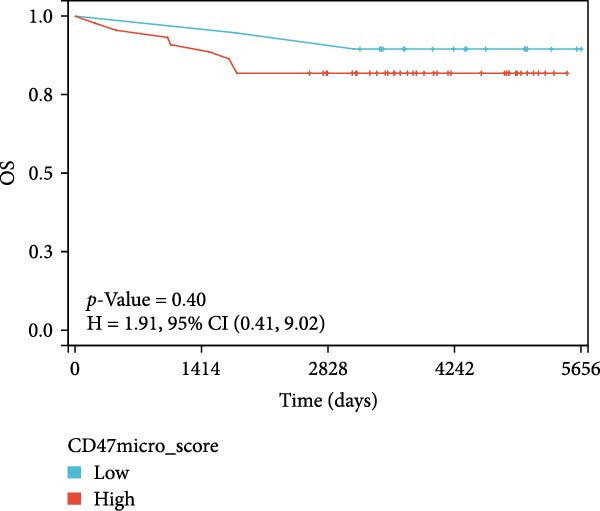
(D)

Figure 4Prognostic impact of combined CD47 and PD‐L1 expression in the CHCAMS Cohort. (A) PFS in combined CD47tumor_score and PD‐L1_TPS. (B) OS in combined CD47tumor_score and PD‐L1_TPS. (C) PFS in combined CD47micro_score and PD‐L1_IPS. (D) OS in combined CD47micro_score and PD‐L1_IPS.

**Figure:**
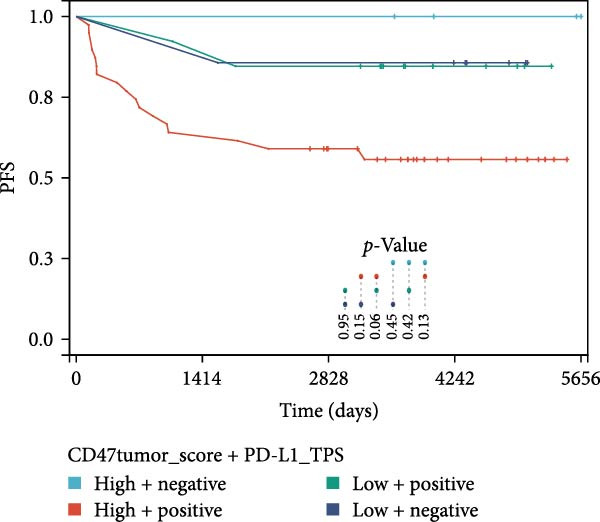
(A)

**Figure:**
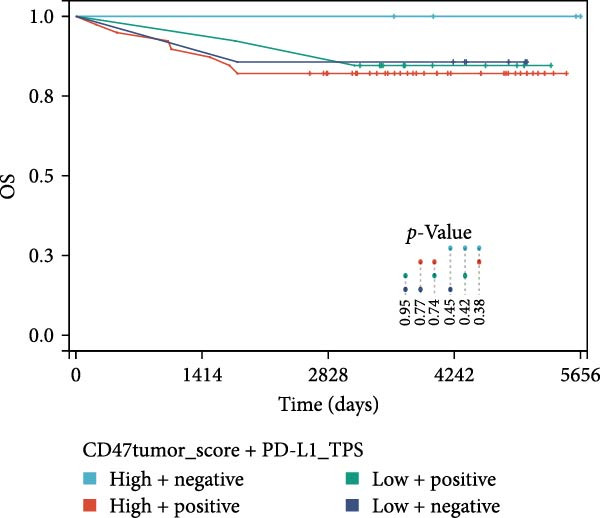
(B)

**Figure:**
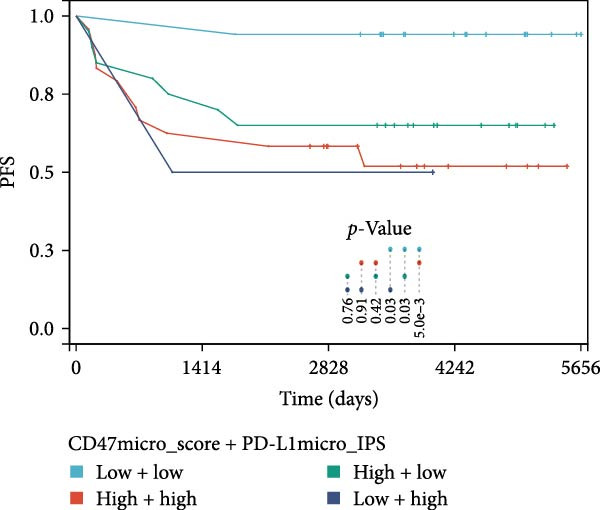
(C)

**Figure:**
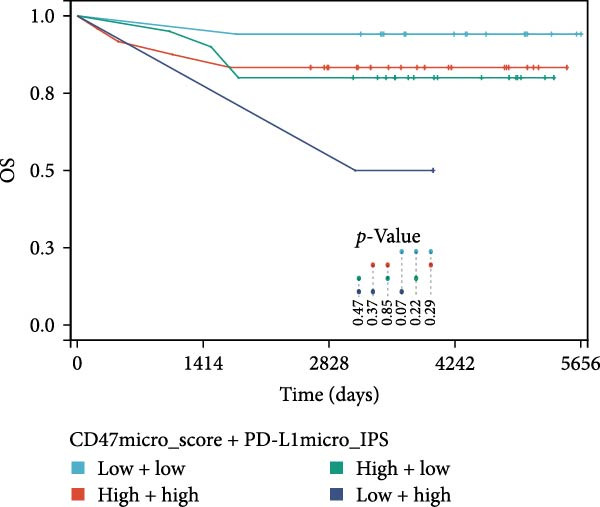
(D)

### 3.4. Public Data and TME Analysis

Next, we retrieved transcriptome profiling data of CHL patients from the GEO database (GSE17920), comprising 130 tumor samples with corresponding prognostic information. The prognostic significance of differentially expressed CD47 in CHL specimens was evaluated. Kaplan–Meier survival analysis and log‐rank tests were conducted to compare survival outcomes between the high CD47 mRNA expression (*N* = 43) and low CD47 mRNA expression (*N* = 87) groups. Consistent with our observations in the internal CHL cohort, patients with high CD47 mRNA expression showed significantly worse PFS (*p* = 0.02), while a trend toward worse OS was observed but did not reach statistical significance (*p* = 0.20) (Figure 5A, B). Differential mRNA expression between the CD47^high^ and CD47^low^ groups was analyzed using R software, identifying 20,824 genes, of which 34 were significantly upregulated differentially expressed mRNAs (DEmRNAs) (Figure S3). KEGG analysis indicated activation of chemokine signaling and cytokine–cytokine receptor interaction pathways (Figure 5C), and GO analysis highlighted granulocyte and neutrophil migration/chemotaxis and myeloid leukocyte migration (Figure 5D), suggesting a proinflammatory immune microenvironment associated with elevated CD47 mRNA expression. CIBERSORT was then applied to estimate immune cell proportions, revealing that the CD47^high^ subgroup in GSE17920 exhibited higher inferred infiltration of M1 macrophages, neutrophils, and eosinophils (*p* < 0.0001, *p* < 0.01, *p* < 0.05; Figure 5E). Collectively, these results indicate that increased CD47 expression is associated with an inflammatory and myeloid‐enriched TME.

Figure 5Association between CD47 mRNA expression and tumor genomic features in BCCA cohort. (A, B) Prognostic values of CD47 in GSE 17920. (C) The upregulated pathways in CHL patients with CD47 high expression. (D) Biological processes related to CD47 high expression in CHL using GO analysis. (E) Differences of Immune cells between CD47^high^ group and CD47^low^ group ( ^∗∗∗∗^ *p* ≤ 0.0001,  ^∗∗∗^ *p* ≤ 0.001,  ^∗∗^ *p* ≤ 0.01,  ^∗^ *p* ≤ 0.05).

**Figure:**
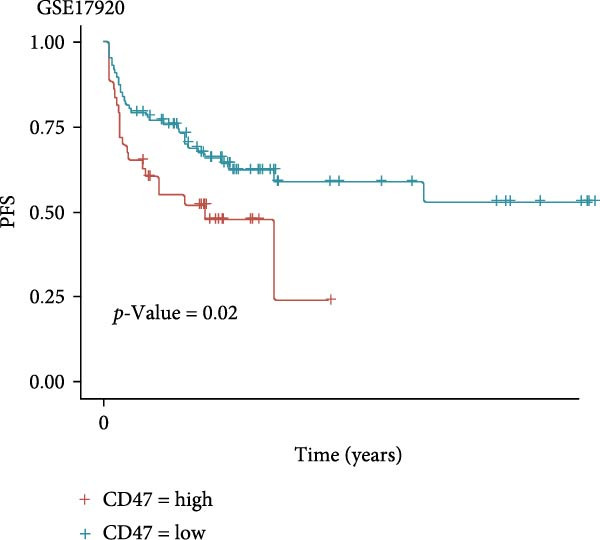
(A)

**Figure:**
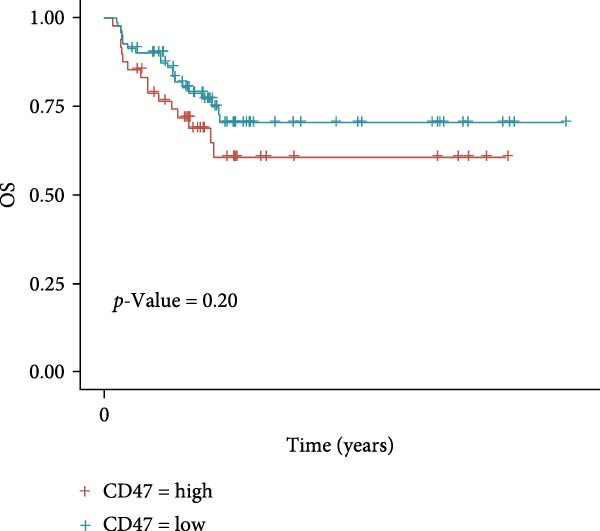
(B)

**Figure:**
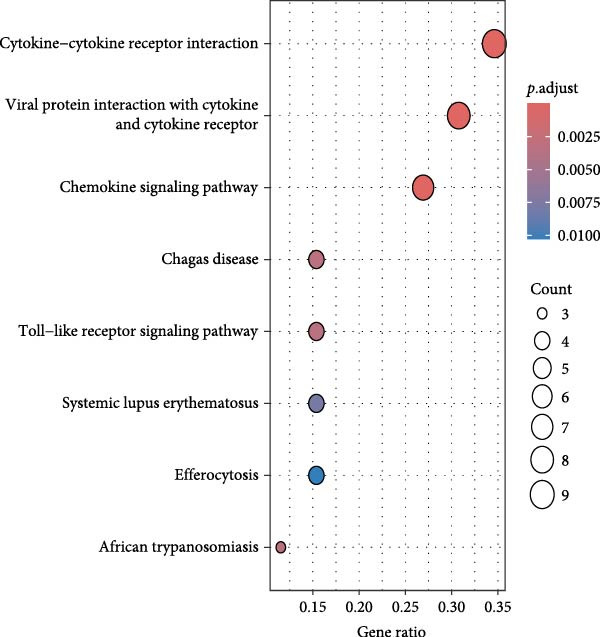
(C)

**Figure:**
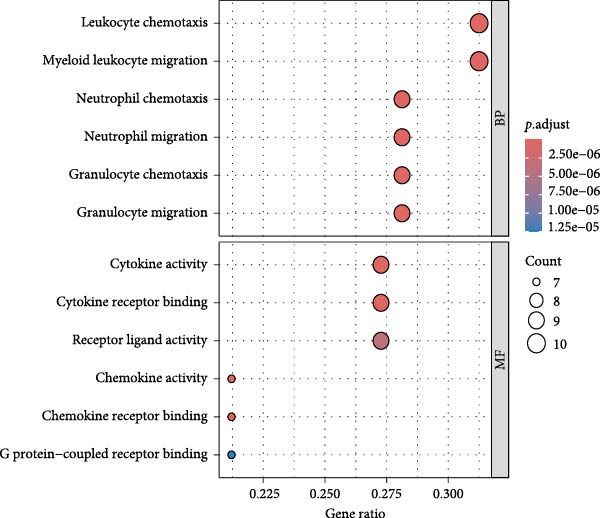
(D)

**Figure:**
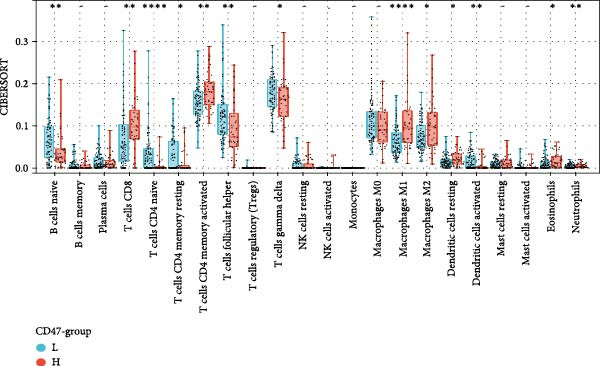
(E)

## 4. Discussion

CHL has the most distinctive cellular composition among lymphomas, including only infrequent HRS cells surrounded by a variety of noncancerous immune and stromal cells, such as various types of T cells, B cells, eosinophils, M1 and M2 macrophages and fibroblasts [20]. With a deeper understanding of cellular interactions between tumor cells and immune cells, the development of novel therapeutic agents, including BV and PD‐1 inhibitors, are now standard‐of‐care treatments in relapsed/refractory CHL [21]. The success of targeting abnormal surface markers has sparked growing interest in developing antibody–drug conjugates to target both tumor cells and other cells in the TME. Herein, our study evaluated the role of CD47 in HRS cells and TME, analyzing its correlation with PD‐1/PD‐L1. Additionally, using publicly available data, we explored the prognostic value of CD47 mRNA expression in the TME of CHL, initially investigated its potential genetic and functional impact on tumor‐associated immune cells.

López‐Pereira et al. [22] first described the prevalent expression pattern of CD47 on HRS cells, our study extended this observation by systematically analyzing its correlation with the immune microenvironment. In our study, CD47 was widely expressed on HRS cells in CHL, and its expression showed no significant correlation with clinical parameters, including gender, EBV status, histological subtype, B symptoms, or clinical stage, consistent with previous reports [11, 22, 23]. Next, our findings that high CD47 expression on either HRS cells or immune cells was associated with inferior PFS, which were in strong agreement with the seminal work by Gholiha et al. [11], who robustly demonstrated in two independent cohorts that CD47 is an independent adverse prognostic factor in CHL. The consistency of this finding across studies firmly establishes CD47 as a key player in CHL pathogenesis and outcome.

Although CD47 expression was not associated with most clinical factors in our study, high CD47 levels on HRS cells were significantly linked to non‐CR (*p* = 0.016), positive PD‐L1 TPS (*p* = 0.012), and higher PD‐L1 IPS (*p* = 0.008). These findings indicated that the prognostic significance of CD47 might depend on the tumor immune microenvironment and had a potentially correlation with PD‐L1. Previous studies have demonstrated a positive correlation between CD47 and PD‐L1 gene expression in several tumor types, including head and neck squamous cell carcinoma, T‐lymphoblastic lymphoma/leukemia (T‐LBL/ALL), and cutaneous T‐cell lymphoma [24–26]. However, the correlation between CD47 and PD‐L1 at the protein level remains controversial. Although significant associations have been observed in several solid tumors, such as non‐small cell lung cancer and hepatocellular carcinoma [27, 28], Yang et al. [24] found a positive correlation between CD47mRNA and PDL1mRNA in T‐LBL/ALL, but no corresponding correlation at the protein level. Likewise, the early study by Gholiha [11] did not find high CD47 expression on HRS cells correlate with SIRP*α* ^+^ leukocytes or the expression of PD‐1, PD‐L1, and PD‐L2 by immunochemistry. A strong MYC signature is a characteristic feature of HRS cells across all CHL histologic subtypes [29]. The previous study in transgenic mouse models and MYC‐induced T‐ALL cell lines showed that MYC regulates CD47 and PD‐L1 mRNA expression by binding to its promoter genes [30]. Therefore MYC‐driven tumorigenesis may coordinately upregulate CD47 and PD‐L1 at the transcriptional level. However, the lack of protein‐level correlation may result from technical differences in heterogeneity of protein expression, detection methods, including different antibody clones and interpretation methods. Our team found that patients with concurrent high CD47 and positive PD‐L1 expression in tumor cells had the poorest survival outcomes. Additionally, CD47 and PD‐L1 expression in the TME demonstrated a combined adverse prognostic effect; patients with high expression of either marker showed significantly shorter PFS. These results suggest that dual blockade of CD47 and PD‐L1 may have synergistic therapeutic benefits.

Then our analyses on the public data, GSE 17920, further verified that patients with high CD47mRNA in CHL exhibited worse prognosis. KEGG analysis highlighted in CD47mRNA high expression CHL was mainly involved in chemokine signaling and cytokine–cytokine receptor interaction pathways, which are known to play a key role in immune evasion and inflammatory responses in various cancers, with chemokine‐receptor interactions influencing TME modification [31]. CD47 may help elucidate the mechanisms and processes underlying these pathways. GO and CIBERSORT analyses presented that neutrophil migration/chemotaxis and myeloid leukocyte migration played a vital role in disease progression, mainly involving immune cells including M1 macrophages, neutrophils, and eosinophils. Neutrophils have been reported to linked with an immunosuppressive function in cancer patients [32], the peripheral blood neutrophil–lymphocyte ratio has been implicated as an independent prognostic factor in many malignancies including CHL, suggesting it as a key component in cancer progression [33]. Barrera et al. [34] found CD47 overexpression might cause an increase in the number of circulating neutrophils by delaying their apoptosis and impairing macrophage‐mediated clearance. Whether the mechanism exists in CHL remains to be explored. Previous studies have revealed that M1 macrophages have a cytotoxic effect and induce malignant cell apoptosis, while M2 macrophages induce apoptosis resistance [35]. Thus, M1 abundance in tumor should be considered as a sign for better prognosis for patients. Shi et al. [13] also reported the prognostic value of M1 macrophages in gastric cancer and demonstrated a positive correlation between CD47 expression and M1 macrophage infiltration. However, they found that M1 infiltration failed to predict patient prognosis within the high CD47 expression subgroup, suggesting that CD47 may promote gastric cancer progression by attenuating the antitumor effects of M1 macrophages [13]. Likewise, in our analysis of GSE17920, patients with high CD47 expression exhibited worse prognosis, together with markedly increased infiltration of M1 macrophages. Moreover, an in vitro study in head and neck squamous cell carcinoma demonstrated that CD47 inhibits phagocytosis by M1 macrophages, rather than by M2 macrophages [36]. These findings highlight the potential prognostic interaction between CD47 expression and tumor associated macrophage populations, which warrants further investigation.

The limitations of the current study merit attention. First, the cohort was heterogeneous, including both limited and advanced stage patients receiving different therapies, which may affect the statistical power of survival analyses. Second, the use of bulk transcriptomic data from public datasets prevents precise cellular attribution of CD47 expression. Third, while CD47 expression in the microenvironment was assessed, its specific cellular sources—such as macrophages or T cells—were not delineated due to the lack of multiplex staining or flow cytometry. Finally, the sample size was relatively small and the study lacks functional validation. Future experiments, such as a larger cohort, in vitro phagocytosis assays, or spatial transcriptomics, are needed to elucidate the independent prognostic value and mechanistic role of CD47 in the CHL microenvironment.

## 5. Conclusion

In this study, we explored the expression and clinical significance of CD47 and PD‐L1 in CHL. CD47 high expression in tumor cells and microenvironment represented an inferior prognosis. CD47 was significantly positively correlated with PD‐L1, patients with simultaneous high expression of CD47 and PD‐L1 had the worst prognosis. The combination of anti‐CD47 and anti‐PD‐1/PD‐L1 therapy treatment is expected to become a novel therapeutic strategy for the treatment of CHL.

## Ethics Statement

This retrospective study was approved by the ethics committee of the Cancer Hospital Chinese Academy of Medical Sciences (No. 2022020714463802). The need for informed consent was waived by the ethics committee of Cancer Hospital Chinese Academy of Medical Sciences. We confirm that all methods were performed in accordance with the relevant guidelines.

## Consent

The authors have nothing to report.

## Conflicts of Interest

The authors declare no conflicts of interest.

## Author Contributions

Xuemin Xue designed the study and revised the manuscript. Xiaoli Feng was involved in research guidance and paper editing. Xiaoyue Xiao performed the statistical analysis, analyzed the data, and wrote the original manuscript draft. Lin Nong interpreted the data and revised the manuscript. Yiyang Luo assisted with collecting the data. Jiyan Dong, Xujie Sun, and Kang Jiang contributed to methodology and manuscript preparation. Xiaoyue Xiao and Lin Nong have contributed equally to this work and share first authorship.

## Funding

This work was supported by grants from the Beijing Hope Run Special Fund of Cancer Foundation of China (Grants LC2021A21 and LC2018L04) and the Cooperation Fund of CHCAMS Beijing & Langfang & SZCH (Grant CFA202502019).

## Supporting Information

Additional supporting information can be found online in the Supporting Information section.

## Supporting information


**Supporting Information** Table S1. Cutoff points of markers by using maximally selected log‐rank statistic. Table S2A. Univariate analyze. Table S2B. Multivariate analyze. Figure S1. Spearman correlation between the CD47tumor_score and the CD47mciro_score. Figure S2. Survival analysis of PD‐1/PD‐L1 expression in the CHCAMS cohort. (A) Kaplan–Meier curves of PFS according to PD‐L1_TPS. (B) Kaplan–Meier curves of OS according to PD‐L1_TPS. (C) Kaplan–Meier curves of PFS according to PD‐L1_IPS. (D) Kaplan–Meier curves of OS according to PD‐L1_IPS. (E) Kaplan–Meier curves of PFS according to PD‐1_IPS. (F) Kaplan–Meier curves of OS according to PD‐1_IPS. Figure S3. Differentially expressed genes analyses. Volcanoes indicating the 34 upregulated mRNAs in CD47^high^ group compared with CD47^low^ group.

## Data Availability

The datasets used and/or analyzed in our cohort are available from the corresponding author on reasonable request.
