# Supplementary material for: CD47 Expression in Classic Hodgkin Lymphoma and Its Association With Tumor Microenvironment
Source: J Immunol Res. 2026 Jan 11;2026:1680256. doi: 10.1155/jimr/1680256 (PMC13140889; doi:10.1155/jimr/1680256)
Supplement: Supplementary file 1 — Supporting Information Table S1. Cutoff points of markers by using maximally selected log‐rank statistic. Table S2A. Univariate analyze. Table S2B. Multivariate analyze. Figure S1. Spearman correlation between the CD47tumor_score and the CD47mciro_score. Figure S2. Survival analysis of PD‐1/PD‐L1 expression in the CHCAMS cohort. (A) Kaplan–Meier curves of PFS according to PD‐L1_TPS. (B) Kaplan–Meier curves of OS according to PD‐L1_TPS. (C) Kaplan–Meier curves of PFS according to PD‐L1_IPS. (D) Kaplan–Meier curves of OS according to PD‐L1_IPS. (E) Kaplan–Meier curves of PFS according to PD‐1_IPS. (F) Kaplan–Meier curves of OS according to PD‐1_IPS. Figure S3. Differentially expressed genes analyses. Volcanoes indicating the 34 upregulated mRNAs in CD47high group compared with CD47low group. [file JIMR-2026-1680256-s001.docx]

**Supplementary Tables**

| Supplementary Table 1 Cutoff points of markers by using maximally selected log-rank statistic | | |
| --- | --- | --- |
| **Markers** | **Cutoff points** | **Statistics** |
| CD47tumor_score | ≥1 | 2.085736 |
| CD47micro_score | ≥4 | 2.462 |
| PD-L1_IPS | ≥30 | 0.6971982 |
| CD47mRNA | ≥9.0563 | 2.141924 |

| Supplementary Table 2A Univariate analyse | | | | | | |
| --- | --- | --- | --- | --- | --- | --- |
| Covariate | Hazard ratio | OS | |  | PFS | |
|  |  | 95% CI | P | Hazard ratio | 95% CI | P |
| CD47tumor_score | 3.2904 | 0.9691 to 11.1717 | 0.0574 | 1.1924 | 0.3104 to 4.5812 | 0.7988 |
| CD47micro_score | 4.9138 | 1.1472 to 21.0469 | **0.0328** | 1.9093 | 0.4084 to 8.9264 | 0.4135 |
| PD-L1_TPS | 2.0867 | 0.2672 to 16.2975 | 0.4853 | 4.9212 | 0.6650 to 36.4170 | 0.1205 |
| PD-L1_IPS | 2.2173 | 0.9082 to 5.4131 | 0.0819 | 1.3361 | 0.3892 to 4.5872 | 0.6469 |
| Clinical efficacy | 3.8674 | 0.8784 to 17.0274 | 0.0752 | 1.2287 | 0.2404 to 6.2793 | 0.8055 |
| Stage | 1.6738 | 0.6391 to 4.3838 | 0.2968 | 0.9354 | 0.1960 to 4.4653 | 0.9336 |

| Supplementary Table 2B Multivariate analyse | | | |
| --- | --- | --- | --- |
| Covariate | Hazard ratio | OS | |
|  |  | 95% CI | P |
| CD47tumor_group | 0.9924 | 0.9539 to 1.0324 | 0.7058 |
| CD47micro_group | 1.0045 | 0.9723 to 1.0377 | 0.7890 |
| PD-L1tumor_group | 2.5740 | 0.2935 to 22.5694 | 0.3958 |
| PD-L1micro_group | 0.9920 | 0.9549 to 1.0306 | 0.6826 |

**Supplementary Figure legends**

**Supplementary Figure 1** Spearman correlation between the CD47tumor_score and the CD47mciro_score

**Supplementary Figure 2** Survival analysis of PD-1/PD-L1 expression in the CHCAMS cohort. (A) Kaplan–Meier curves of PFS according to PD-L1_TPS. (B) Kaplan–Meier curves of OS according to PD-L1_TPS. (C) Kaplan–Meier curves of PFS according to PD-L1_IPS. (D) Kaplan–Meier curves of OS according to PD-L1_IPS. (E) Kaplan–Meier curves of PFS according to PD-1_IPS. (F) Kaplan–Meier curves of OS according to PD-1_IPS.

**Supplementary Figure 3** Differentially expressed genes analyses. Volcanoes indicating the 34 upregulated mRNAs in CD47high group compared with CD47low group.

**
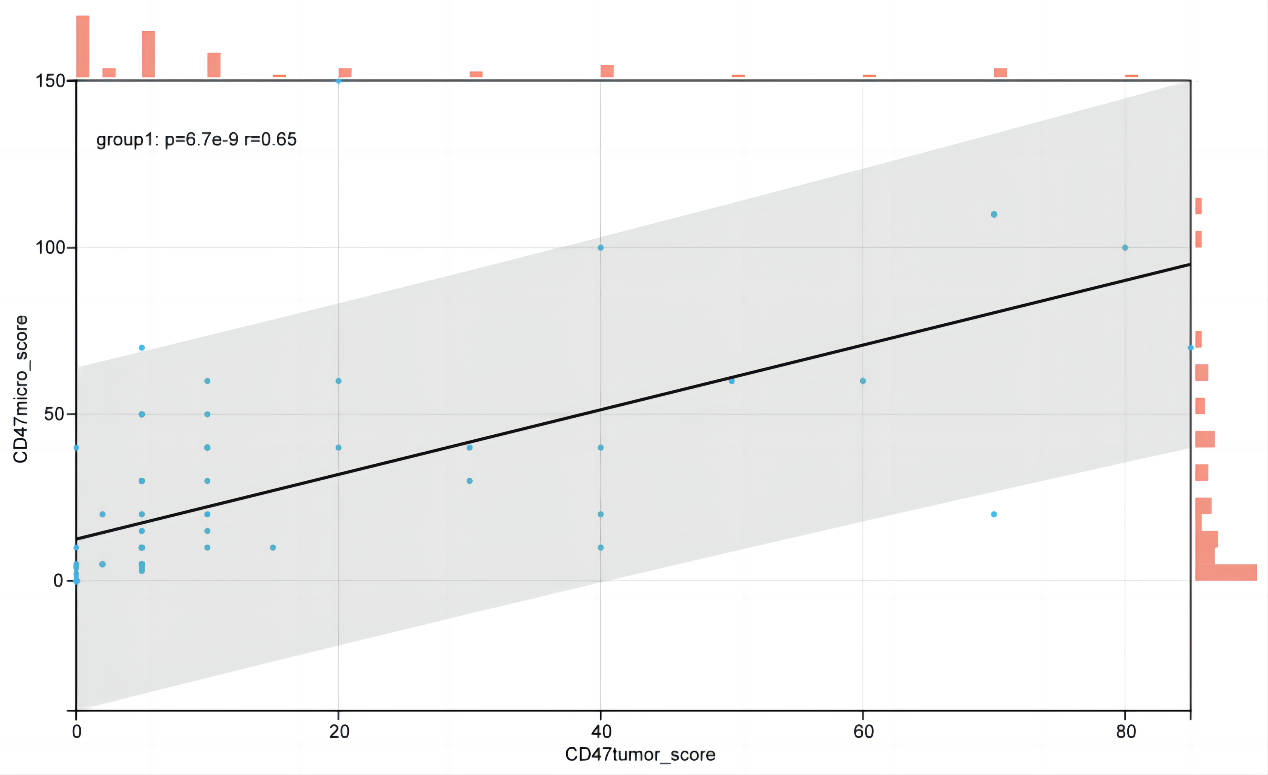
**

**Supplementary Figure 1**

**
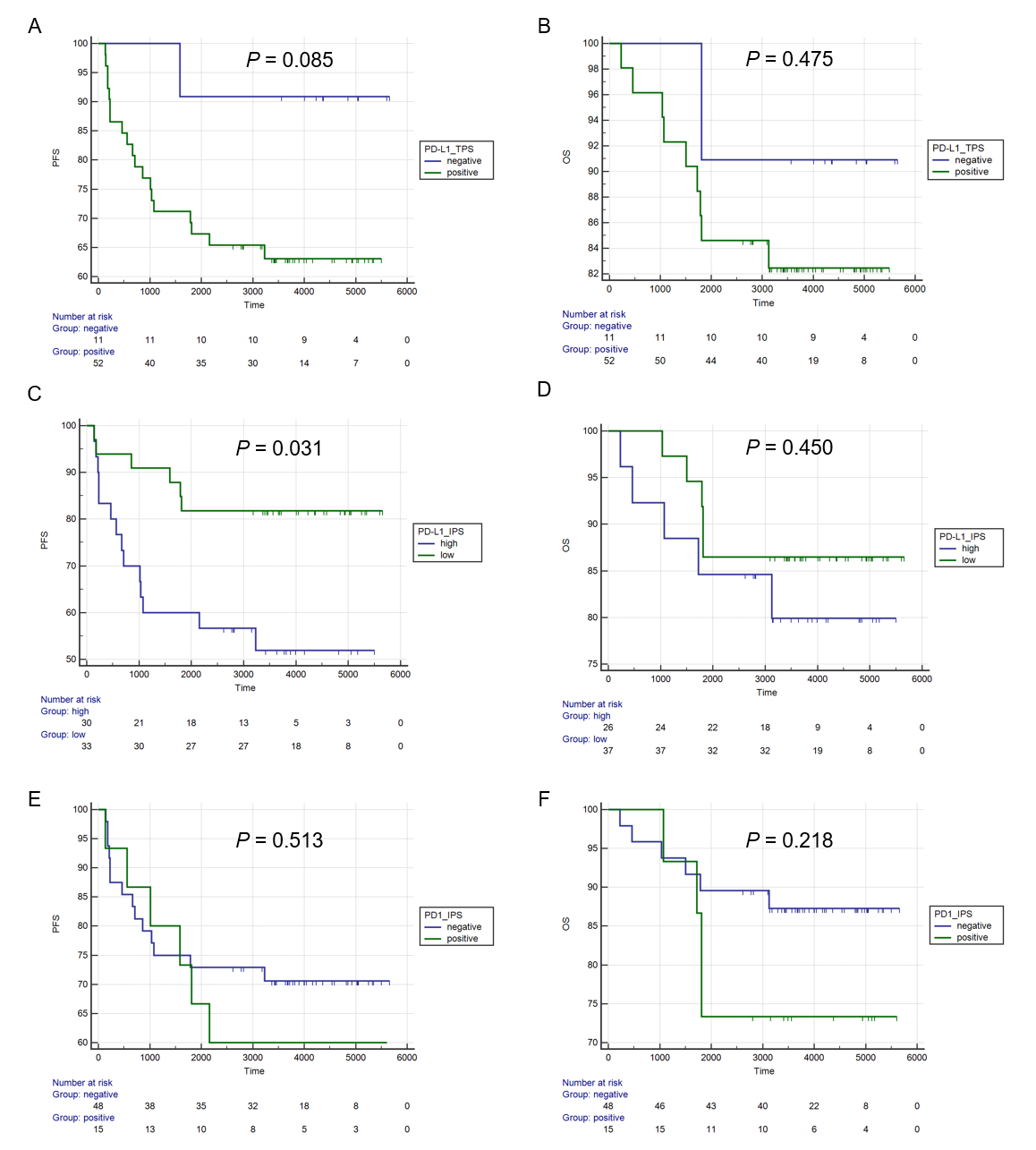
**

**Supplementary Figure 2**

**
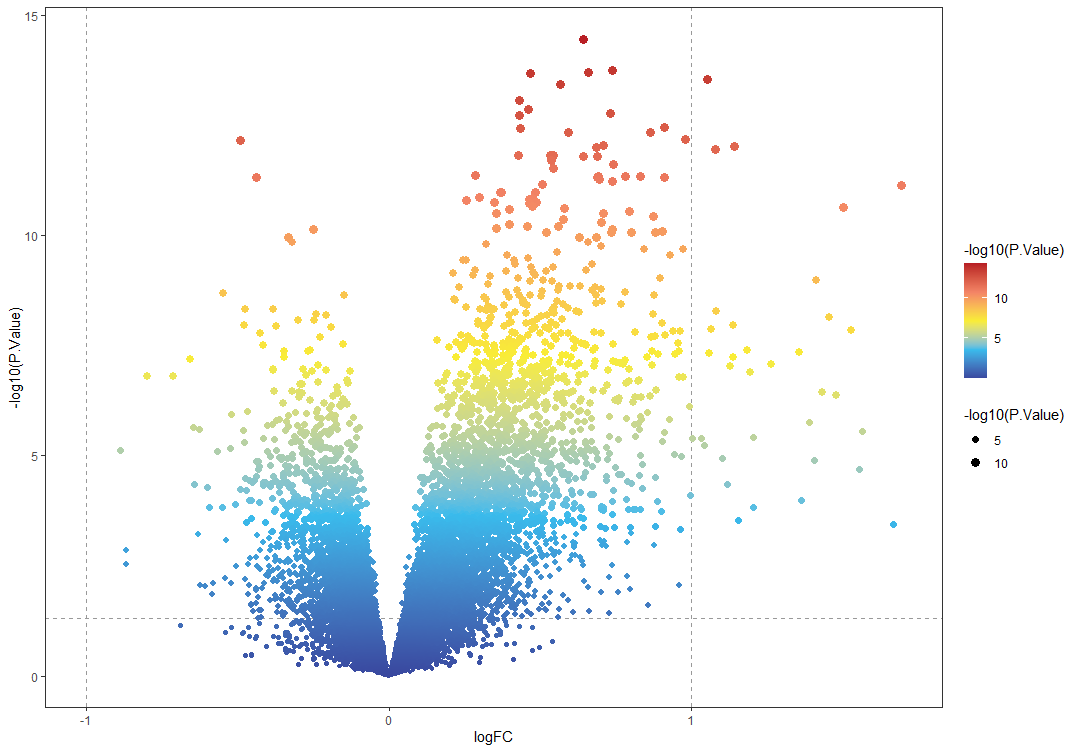
**

**Supplementary Figure 3**
